# Human Factor Engineering Research for Rehabilitation Robots: A Systematic Review

**DOI:** 10.1155/2023/2052231

**Published:** 2023-02-06

**Authors:** Duanshu Song, Songyong Liu, Yixuan Gao, Yuexin Huang

**Affiliations:** ^1^School of Mechatronic Engineering, China University of Mining and Technology, Xuzhou 221116, China; ^2^School of Mechatronic Engineering, Jiangsu Normal University, Xuzhou 221116, China; ^3^Key Laboratory of Industrial Design and Ergonomics, Ministry of Industry and Information Technology, Northwestern Polytechnical University, Xi'an 710072, China; ^4^School of Industrial Design Engineering, Delft University of Technology, Delft 2628CE, Netherlands

## Abstract

The application of human factors engineering for rehabilitation robots is based on a “human-centered” design philosophy that strives to provide safe and efficient human-robot interaction training for patients rather than depending on rehabilitation therapists. Human factors engineering for rehabilitation robots is undergoing preliminary investigation. However, the depth and breadth of current research do not provide a complete human factor engineering solution for developing rehabilitation robots. This study aims to provide a systematic review of research at the intersection of rehabilitation robotics and ergonomics to understand the progress and state-of-the-art research on critical human factors, issues, and corresponding solutions for rehabilitation robots. A total of 496 relevant studies were obtained from six scientific database searches, reference searches, and citation-tracking strategies. After applying the selection criteria and reading the full text of each study, 21 studies were selected for review and classified into four categories based on their human factor objectives: implementation of high safety, implementation of lightweight and high comfort, implementation of high human-robot interaction, and performance evaluation index and system studies. Based on the results of the studies, recommendations for future research are presented and discussed.

## 1. Introduction

Stroke is a cerebrovascular disease that causes significant morbidity, disability, and mortality, often leading to motor dysfunction or permanent disability [1, 2]. With the increasing ageing of the population, stroke has become the major cause of physical disability worldwide [3], and other chronic diseases, such as diabetes and arthritis, also usually have a high rate of disability [4]. These factors have led to an even faster increase in the number of patients with motor dysfunction. Research in rehabilitation medicine has shown that timely intervention of effective rehabilitation measures in the early stages of the disease can help patients to improve and rebuild their motor functions. As a result, the market for the rehabilitation needs of people with motor dysfunction will continue to expand. However, traditional rehabilitation methods mainly rely on therapists' experience, making it challenging to meet the requirements of high-intensity and repetitive training [5]. Moreover, there is a shortage of rehabilitation therapists, rehabilitation platforms, and facilities and a huge gap in the supply of rehabilitation institutions and services [6]. Therefore, safe, reliable, and efficient rehabilitation technologies are needed to alleviate the current imbalance between supply and demand in the rehabilitation market. With its advantages of high intensity, high precision, and high efficiency, the development of rehabilitation robots, which combine clinical rehabilitation medicine with robot-assisted rehabilitation technology, has opened up new technological avenues for improving stroke or postoperative rehabilitation and has grown to be an essential way to meet the demand for rehabilitation and ageing services in the future [7–11].

Rehabilitation robotics is a specific branch that focuses on helping patients restore or reestablish motor function. Rehabilitation robotics can be used in many aspects of physical therapy. It attempts to combine multiple technologies to maximize the physical rehabilitation training needs of patients, making extensive advances in robotic prostheses and other fields [12]. Thus, in numerous fields, rehabilitation robots have become a recent hotspot for research applications both domestically and internationally. At this stage, the research on rehabilitation robots mainly focuses on technical challenges, such as the lightweight and flexible design of materials, motion space algorithms, and virtual reality applications [13]. However, with the development of the rehabilitation market, the humanized aspects of rehabilitation technology need to be continuously optimized and refined to make rehabilitation training safe, reliable, and efficient.

The direct target of rehabilitation robots is the patient, and human factors goals such as safety, comfort, reliability, and adaptability are key considerations. Some rehabilitation products, such as HAL-5, Lokomat, and Hero Arm, have entered the market [14]. But in terms of the overall rehabilitation robot market, in addition to higher manufacturing costs, human factors, such as structural human-robot fit, motion flexibility, wearing comfort, ease of operation, individual compatibility, pathological applicability, safety, and reliability, are still far from safety, comfort, and efficiency goals. Therefore, there is still a gap between the goal of safe, comfortable, efficient, and quality rehabilitation services [15], which brings a challenge to the human factor engineering research of rehabilitation robots. Applying human factor engineering theory to rehabilitation robotic systems can find out human-robot interaction problems in these systems. Moreover, human factor engineering applied to rehabilitation robotic system can creatively propose targeted solutions to remedy the deficiencies, which is the key to making the human-robot system more suitable for 'patients' physiological and psychological characteristics and to solving the existing human factors problems of rehabilitation robots.

The above-given context raises the question of how far the research application of human factor engineering in rehabilitation robotics has progressed. In the field of rehabilitation robotics, review articles [16–18] can provide a systematic review and general analysis of the framework and progress of current research, which is a high concentration and sublimation of valid information and is pivotal to the research progress and development trend of the field. To the best of our knowledge, many review papers have been published on rehabilitation robotics. However, few review papers systematically review the progress of rehabilitation robotics with human factor engineering as the research point of view. In addition, there is no discussion on the main issues and future directions of human factor engineering research structures in rehabilitation robotics to enhance the humanized design of rehabilitation devices by covering different connotations of human factors goals. Therefore, this study aims to supplement the academic literature, comprehensively sort out the current human factors research directions and the main points in the field of rehabilitation robots, address the shortcomings of existing human factors research frameworks and technical solutions, provide constructive guidance for future research directions, and accelerate the process of humanized design of rehabilitation robots.

To this end, this study focuses on the specific connotation of human factors goals, analyzes and composes the four human factors goals currently focused on research from the perspective of the system level and characteristics, explores the shortcomings of existing human factors goals and research contents and technical solutions in terms of research depth and breadth, and provides supplements and suggestions for further research contents. This study is crucial for further securing and improving the effect of stroke or postoperative rehabilitation treatment and enhancing the human-robot interaction experience. In the long run, this work can also improve the quality and overall level of rehabilitation medical equipment and help the development of rehabilitation equipment iterations.

In this regard, this study has the following contributions in various aspects: (1) the research progress of robotic rehabilitation systems is analyzed from a brand new perspective, such as human factor engineering, and this is used as an innovation and entry point to analyze and sort out the four human factors objectives that are currently the focus of research. (2) The importance of human factor engineering in rehabilitation robotics research is clarified by highlighting the motivation and challenges of improving the adaptability and user experience of rehabilitation robots through human factor engineering research. (3) Suggestions are offered from the perspectives of the connotation categories of human factors goals and key technology solutions to provide a theoretical foundation and a direction for future study. (4) The results of various datasets and available sources are explained to support the classification of human factors goals, and tables are provided comprising all relevant datasets.

This study is organized as follows.  Section 2 addresses the methodological process applied throughout the study, including the literature search, screening, quality assessment, and data analysis.  Section 3 presents the findings of the study in four areas. Based on this, recommendations for future research are presented and discussed in Section 4.  Section 5 concludes the paper.

## 2. Methodology

In order to address our research topic, we conducted a systematic review of the literature related to this topic, drawing on the methodology and process of Bal et al. In the study of [19], a literature review on head-mounted displays and their working content are described.

### 2.1. Search Strategy

A systematic search was conducted to collect and summarize all research on human factor engineering in rehabilitation robotics as the research topic. We searched for the literature in three steps. First, databases, such as Web of Science, ScienceDirect, IEEE, EI, SCOPUS, and SpringerLink, were selected and searched, with the database time limit set from January 2011 to May 2022. These databases were systematically searched using Boolean algebra. Three columns of search terms were freely combined: the first and second columns were synonymous with rehabilitation robotics and human factor engineering, respectively. The third column of search terms is a paraphrase of the “human factor engineering” connotation due to the broad scope of “human factor engineering.” A total of 510 Boolean-configured search terms were composed and examined in each database. A complete view of the search terms can be found in Table 1. Second, the references of the selected studies were scanned to identify further relevant literature. Third, we checked the articles citing the selected studies through Google Scholar.

### 2.2. Selection Criteria

Studies that met the following selection criteria were included in the review, and studies that failed to meet any of the following criteria were excluded:

1. The studies need to be empirical and examine the human factor engineering research component of rehabilitation-oriented robots. For this purpose, quantitative, qualitative, and mixed methods studies were considered.
2. The research needs to involve research that enhances the human factors suitability of rehabilitation robots, such as safety, comfort, light weight, human-robot relationships, task allocation, the naturalness of human-robot interaction, and system performance evaluation. The research also needs to include technical solutions and measures.
3. The research needs to be an academic paper. However, as the emerging field of human factor engineering research in rehabilitation robots is thus far in its infancy, we decided to expand the journal research to include book chapters and conference papers.

### 2.3. Analysis Methods

#### 2.3.1. Quality Assessment

Following the initial screening, the subject staff critically assessed qualitative (Table 2), quantitative (Table 3), and mixed methods studies (Table 4) using QARI, EPHPP, and MMAT, respectively, with the authors resolving differences in selection through discussion. IBM SPSS Statistics 26 was used to measure interrater reliability, and the intragroup correlation coefficients were high, i.e., 0.80 for qualitative studies, 0.91 for quantitative analyses, and 0.82 for mixed methods studies.

#### 2.3.2. Data Extraction

Data extraction was carried out in two main ways. First, the essential characteristics of the final included studies were sorted to include the date of publication, the study's country, and the literature type. Correspondingly, the research design and data collection methods used in the selected studies were summarized, and information on the study participants was added to count the number of participants in the study. Second, under the human factor engineering connotations category, we report the human factor engineering research components involved in the studies reviewed, using specific human factors targets as the basis for classification.

#### 2.3.3. Overview of Results

We found that in human factor engineering research on rehabilitation robots, the achievement of the same research goal maps to multiple dimensions of rehabilitation robot system design; similarly, the design of the same dimension of a rehabilitation robot system affects the achievement of multiple human factors embedded goals under the category of human factor engineering. To provide a clear and intuitive overview of the results of human factor engineering research on rehabilitation robots, the results of the data analysis have been reviewed using the former classification approach. This approach summarizes and integrates the current state of research on each human factors goal within the human factor engineering research scope of the selected studies in a multifactor and multimethod classification of one human factors goal.

## 3. Results

### 3.1. Findings of Reviewed Study Characteristics

**Figure 1:**
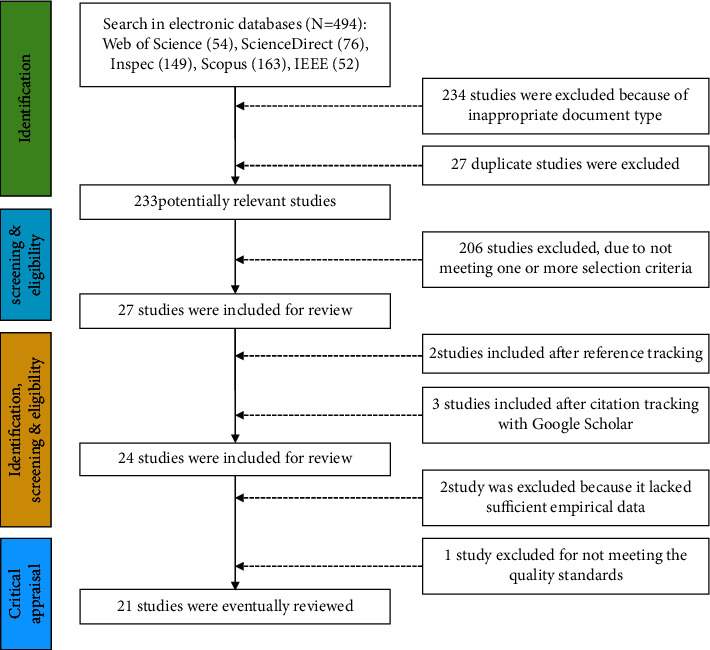
Flow chart of the literature search and screening.

This study used the style of systematic reviews and meta-analyses guidelines illustrated in Figure 1, [20, 21]. The researcher independently searched the above-given databases according to the strategy and obtained 494 relevant studies from the initial review, and two were included by reading references to relevant articles. Two researchers familiar with human factor engineering research on rehabilitation robots and related evaluation tools screened and analyzed the literature. The entire process was blinded to the inclusion criteria. Studies in which there was difficulty making a decision were discussed and negotiated with a third researcher before being identified. Finally, 21 items were included (Figure 1).

**Figure 2:**
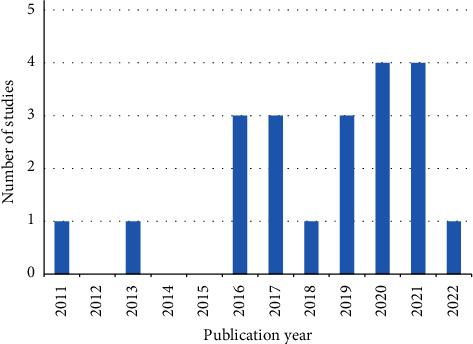
Publication year and the corresponding number of relevant studies.

Table 5 presents an overview of the essential characteristics of the final included literature, including elements of characteristics such as author, year of publication, type of literature, study methodology, and participant information. The data for the year of publication of the selected studies show a large number of early studies in the period 2011–2016, and a sharp increase in the number of studies published each year since 2016. This indicates that human factor engineering in rehabilitation robotics is gradually becoming a research hotspot (Figure 2).

In addition, 10 studies were quantitative, 2 were qualitative, and 9 were based on mixed methods. All of these studies were experimental. 18 studies used actual experiments, basically choosing healthy people or rehabilitation patients with motor dysfunction as experimental subjects; 7 studies used virtual simulation in the form of experiments, of which 4 studies used a combination of both simulation and actual experiments and controls, while none of the more subjective research methods, such as questionnaires, were involved.

Table 6 summarizes and analyses the human factors targets in the included studies, including safety (2 studies), lightness and comfort (4 studies), suppleness and stability (7 studies), coordination (2 studies), adaptive and on-demand assistance (2 studies), and performance evaluation (15 studies). The performance evaluation involves safety, comfort, motor performance, and ergonomics and relies mainly on evaluation indicators that have not yet developed a uniform standard.

### 3.2. Findings of the Reviewed Study Results

We found that in human factor engineering research on rehabilitation robots, the achievement of the same research goal maps to multiple dimensions of rehabilitation robot system design; similarly, the design of the same dimension of the rehabilitation robot system affects the achievement of multiple human factors embedded goals under the category of human factor engineering. To provide a clear and intuitive overview of the results of human factor engineering research on rehabilitation robots, the results of the data analysis are reviewed using the former classification approach. This approach summarizes and integrates the current state of research on each human factors goal within the scope of human factor engineering research on rehabilitation robots in the selected studies in a multifactor and multimethod classification of one human factors goal.

#### 3.2.1. Realization of High Safety

As a typical human-robot interaction device, safety design is a primary consideration. There are two central safety control schemes in everyday use today: an external control system scheme based on external monitoring and an internal control system scheme based on the robot's body design. Pan et al. [22] designed a safety supervisory fuzzy controller (SSFC) based on the impaired limb's real-time physical state by extracting and recognizing the impaired limb's tracking movement features. The proposed SSFC was used to automatically regulate the desired force either to account for reasonable disturbance resulting from pose or position changes or to respond adequately to an emergency based on an evaluation of the impaired limb's physical condition. The results show that the proposed approach is practical for achieving safety and robustness in rehabilitation robots. Li et al. [23] investigate the range of stiffness of flexibly compliant joints based on a compromise between the nonlinear model of human-robot collision and the static equilibrium conditions of the rehabilitation robot, taking into account both safety and motion performance requirements to improve the safety factor of use.

#### 3.2.2. Realization of Light Weight and High Comfort

There is still room for weight reduction in the quality of existing rehabilitation robots, and portability and wearability need to be improved. In recent years, research has increased into reducing the structural weight of rehabilitation robots and enhancing wearer comfort.

Using lightweight composite materials plays an active role in reducing the structural weight of rehabilitation robots and improving the wearing comfort. For example, using materials such as polyethene and polypropylene can significantly achieve lightweight designs for rehabilitation robots. One study [43] reduced the mass of an exoskeleton to only 1.3 kg by using a flexible neoprene material, which significantly improved portability and wearing comfort. Liu et al. [24] contributed to the lightweight development of a rehabilitation exoskeleton based on 3D printing technology and virtual simulation. The printing material was polylactic acid with a 50% print fill. Apart from the servo motor (1.5 kg) and planetary reducer (1 kg), the total structure weight was 0.53 kg and the total exoskeleton weight was 3.03 kg. The excellent weight reduction and improved comfort and portability also demonstrated that the 3D-printed exoskeleton rehabilitation robot could output appropriate joint torque for progressive resistance training of the upper arm.

Weight reduction can also be achieved by simplifying complex structures and softening rigid structures. In addition, it has been found that using the principle of gravitational balance to balance some of the weight in terms of structural simplification makes the exoskeleton simple, lightweight, and well-followed, minimizing the physical footprint while maximizing mechanism isotropy and device functionality [44, 45]. Ann Witte et al. [25] used a robust and lightweight frame and showed that the exoskeleton weighed only 0.76 kg and demonstrated that the plate carbon fibre frame was able to accommodate inward/outward rotation of the knee and was sufficiently pliable to flex inward and outward to provide a good fit for legs of different diameters. The compliance of the straps and soft tissues improves the comfort of use and may mitigate increases in step width and circumduction.

The rational weight distribution configuration, i.e., improving the drive layout and optimizing the drive method, can also achieve lightweight and comfort goals. Some studies have reduced the total weight by using a cord and pneumatic drives [46–49] while optimizing the weight distribution [50–53] to achieve weight reduction and increased wearing comfort. Wang et al. [26] further demonstrated optimizing the configuration layout by optimizing the shoulder strap connection configuration and suit layout using a rolling knee joint, a double hinge mechanism, and a low impedance mechanical drive, using lightweight materials and simplified structures effectively reduced weight and minimized misalignment between the robot and knee joints, and greatly enhancing the comfort of the human-robot interaction.

Flexible exoskeleton robots can be said to combine all three of these aspects: eliminating the rigid frame of traditional exoskeletons, optimizing the actuation methods (often using motors, cords, and pneumatic drives) based on the use of lightweight materials [54–58], and allowing for good wearing comfort and safety and have become a rapidly developing field of increasing importance in assistive, disability and rehabilitation training [59–64]. Furthermore, in terms of comfort, Gao et al. [27] looked at bionic structures and human-robot coupling to improve the human-robot coupling of rehabilitation robot configurations and the compatibility of movements, thus enhancing the comfort of human-robot interaction.

#### 3.2.3. Realization of High Human-Robot Interactivity

A rehabilitation robot is a human-robot collaborative intelligence system with a human core. Good human-robot interaction performance includes flexibility, coordination, adaptivity, on-demand assistance, and pathological adaptability of rehabilitation movements, all of which are prerequisites for efficient human-robot collaboration. Many factors affect human-robot interaction performance, including human-robot interaction control, anthropomorphic structure, human-scale adaptability, motion planning, and many other aspects, mainly involving control systems, mechanical systems, software systems, and others.

**Figure 3:**
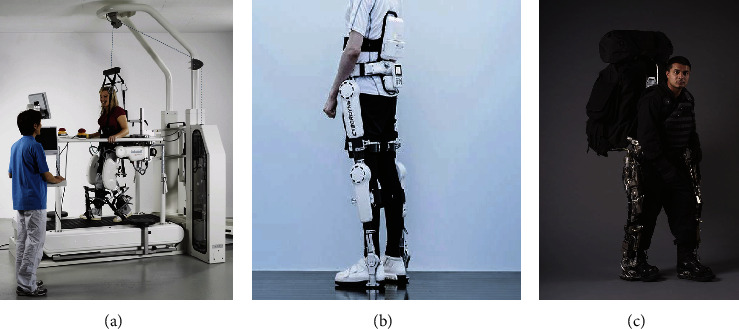
Wearable robot lokomat (a), HAL (b), and BLEEX (c).

The suppleness of human-robot interaction is influenced by the interaction control, but it also focuses on three main aspects mapped on the drive mechanism, structure, and interface connection components of the rehabilitation robot [65]. Regarding interaction control, impedance control, electromyography (EMG) control, and brain-computer interface (BCI) control are more commonly used. In addition, some researchers have also investigated acoustic control [66] and master-slave control [67]. The object of impedance control is the dynamic relationship between the human-robot interaction force and position. Its outstanding advantage over hybrid force/position control is its ability to modulate the suppleness of rehabilitation movements and make mechanical joints exhibit anthropomorphic dynamic properties. Early studies by Lokomat, ALEX, HAL, and BLEEX [68–71]: all used impedance control to achieve the flexibility of the rehabilitation motion, as shown in Figure 3. Four studies delved into the flexibility and stability of the rehabilitation motion, of which Chen et al. [28] and Wang et al. [29] combined impedance control, motion planning trajectory tracking, etc. The studies were conducted to improve the trajectory tracking accuracy and robustness to achieve the supple stability control of the rehabilitation robot.

Drive mechanisms are essential for realizing flexibility, with the series elastomeric actuator (SEA) becoming the most popular drive option [72]. The SEA is characterized by an elastic element with a fixed stiffness connected in series with a motor or motor block and placed before the actuator load [73, 74]. Compared to rigid actuators, using SEAs has shown better performance in six areas: human-robot interaction, safety, energy efficiency, vibration resistance, and reversibility [75–79]. The deformation of elastic components can also be used to measure joint torque, thereby reducing the need for force sensors [80]. Furthermore, despite their reduced bandwidth, SEAs exhibit better torque tracking during exoskeleton walking when operating in exoskeleton experiments [72, 81].

Li et al. [30] proposed a real-time parallel variable stiffness control method by analyzing the SEA interface, discussing the limiting factors of impedance frequency, and combining both safety and high-performance SEA with a cascaded impedance controller with a stiffness adjustment regulator, thus achieving real-time stiffness adjustment of the stiffness and maintaining a certain level of flexibility stability. In addition, the cable drive positively impacts the achievement of flexibility [82]. Wang et al. [31], on the other hand, used flexible drive materials, such as cables and investigated a cable tension control method for a cable drive unit (CDU) loading system to improve flexibility and reduce the robot's impact on the internal tension of the cable, which was adjusted to improve flexibility and reduce the severe impact of the robot on the human body. Moreover, the small contact area between the human body and the robot reduces the human-robot motion interference caused by external interference, thus enhancing the flexibility of the human-robot interaction.

The variation in the rehabilitation solutions required by different patients or the same patient at different stages of rehabilitation places a demand on adaptive on-demand assistance for robotic rehabilitation systems. Adaptive control strategies can provide many benefits for exoskeletons, as the controller can automatically adjust for the variability between each patient and the changing needs of individual patients. Adaptive control of rehabilitation exoskeletons is currently immature [83]. Two studies have addressed adaptive on-demand assistance. Hussain et al. [32] developed an adaptive seamless assist-as-needed (AAN) control scheme based on the robust CRVC law as the primary position controller and a robust adaptive control method that can provide seamless adaptive assistance based on the pathology stage. Li et al. [33] realized a personalized lower limb rehabilitation robot mechanism with different manoeuvrability and movement patterns by comparing the effects of different lower limb movement patterns on human muscle activity based on three different driving modes and human-robot coupling models for rehabilitation robots.

The coordination of rehabilitation movements is based on precisely recognizing the patient's movement intentions. EMG signal control is noninvasive, flexible, precise in the patient's movement intentions, and highly operable and safe. At present, the application of surface electromyography signals in rehabilitation robotics has been increasingly studied [84–87], such as the relationship between surface electromyography (sEMG) signals and muscle force, the relationship between EMG signals and joint torque, and the relationship between EMG signals and the kinematics of the limb during free movement in space and others and has achieved better control. The results were good.

EMG-based control methods can usually be divided into two types: neuro-fuzzy control methods based on EMG (i.e., a combination of fuzzy control methods and neural network control methods) and muscle model-based EMG control methods, in which a matrix is associating the user's joint torque with a specific muscle is used to obtain the boost torque [88]. For the latter, Xie et al. [34] found that the human-robot coupling (HRC) torque was equal to the motor torque of the robot and the muscle torque of the human arm, established a human-robot coupling dynamics model and solved for dynamic parameters through HRC dynamics analysis to achieve coordination of rehabilitation movements through more accurate dynamics control of the upper limb rehabilitation robot. Gui et al. [35] combined a central pattern generator (CPG) network into the one-dimensional joint spatial conductance control of a customized lower limb robotic exoskeleton with four degrees of freedom. The subject's unilateral knee torque was detected based on the corresponding muscle EMG signal. The torque is transformed into an additional set of state variables for CPG based on the one-dimensional admittance controller. CPG harmonically adjusts the predefined trajectories by the additional state variables to ensure coordinated movements and safety for the user.

#### 3.2.4. Performance Evaluation Studies

Effective performance evaluation methods and indicators are the basis for optimizing the performance of human-machine equipment. A total of eight papers deal with performance evaluation studies, mainly focusing on ergonomic comfort, stability of muscle activity and movement, compatibility, fit, and other aspects of human-robot effectiveness performance evaluation.

Safety is a critical evaluation index for human-robot interaction devices and is an essential prerequisite for transforming experimental results into clinical applications. The safety evaluation of rehabilitation robots aims to achieve safe interaction between patients and rehabilitation robots and currently focuses on the safety performance of the rehabilitation robot system itself during human-robot interaction. Wang et al. [36] defined safety performance factors, such as rope tension, system stiffness, fluctuation of motion velocity of slider B1 in the rigid motion support chain, and motion velocity of the lower limb traction point by mechanical analysis of the bionic muscle cable-driven lower limb rehabilitation robot (BM-CDLR) and gave a structural safety evaluation index and used the BM-CDLR safety evaluation index by combining the velocity influence function.

As a wearable device that acts directly on the surface of the human body, comfort is the critical performance of human-robot interaction. The comfort of rehabilitation robots is mainly evaluated in terms of their appearance, materials, operational comfort, and wear fatigue. The main methods for evaluating the comfort of rehabilitation robots are subjective evaluation and objective experimental methods, with subjective evaluation usually taking the form of questionnaires and objective evaluation mainly taking the form of pressure distribution and physiological electrical signal experiments.

Two studies dealt with comfort evaluation. De Rossi et al. [37] applied distributed pressure sensors between the user and the exoskeleton to assess the comfort of interactive training with rehabilitation robots by providing accurate, redundant, and reliable measurements of the distribution of interacting forces. Grazi et al. [38], on the other hand, performed a reverse muscle fatigue perspective for comfort evaluation by identifying evaluation metrics, such as electromyography (EMG), heart rate, and subjective user feedback, to evaluate comfort utilizing objective physiological experiments and personal user perception recordings.

There is no unified index system for evaluating human-robot efficacy performance. Four studies evaluated the motion performance of rehabilitation robots, such as matching, compatibility, and stability, and one involved the evaluation of human-robot ergonomics. Zhang et al. [39] and Di Natali et al. [40] evaluated the motion performance broadly. However, the evaluation metrics differed, with the former using metrics such as motion isotropy and condition number and the latter using metrics such as joint angular trajectory, range of motion (ROM), velocity, and angular velocity. Tsuji et al. [41] assessed the matching of human-robot motion based on position and force information. By using dynamic programming (DP) to match the force and position information recorded by the rehabilitation robot, they demonstrated that the motion matching technique with the addition of force information based on the motion information as the eigenvalue could improve the matching motion accuracy in the case of active and passive motion. Li et al. [42] recorded the interaction forces, torques, and displacements at the connection interface in static and dynamic modes and found that the results in static mode could be used to assess the conformation and size of the exoskeleton for the user's physical adaptation. In contrast, the results in the dynamic model were used to assess the human-robot motion compatibility of the exoskeleton.

## 4. Discussion

In this study, we conducted a systematic review of human factor engineering research on rehabilitation robots and classified the human factors research content from the perspective of human factors objectives. The research mainly focuses on human factors objectives and content, such as safety, weight and comfort, human-robot interaction, and performance evaluation of rehabilitation robots.

Research on human factor engineering in rehabilitation robots has already achieved some milestones, which not only solves many drawbacks, such as low efficiency of rehabilitation by traditional rehabilitation methods but also achieves high efficiency, high precision, and personalized rehabilitation treatment to a certain extent. Therefore, applying human factor engineering in rehabilitation robots is a necessary technical means to achieve effective rehabilitation treatment in the future. However, some critical human factors issues regarding safety, lightness and comfort, human-robot interaction, and performance evaluation have not yet been carried out or require in-depth research. On the basis of an analysis of the aforementioned human factor engineering research for rehabilitation robots, it is proposed that safety protection mechanisms, individual difference compatibility mechanisms, human-robot interaction incentive mechanisms, and human-robot system evaluation indicators and systems can be further developed in future research.

### 4.1. Safety Protection Mechanisms

The safety design of the rehabilitation robot should include two levels: (1) the rehabilitation robot and the human being are in the same movement space to jointly complete the corresponding task, and it is necessary to prevent the robot from colliding with the human body to ensure the user's safety. (2) The safety of the rehabilitation training mode, intensity, and range of motion for the patient's current disease condition. In terms of safety protection mechanisms, most of the current safety research focuses on the internal control system designed by the rehabilitation robot itself, with little research involving the external control system between human and robot and a lack of safety assessment of the functional parameters of rehabilitation training for the patient's pathology.

To achieve multiple protections for patient safety, future research should focus on two critical aspects of the rehabilitation robot: mechanism design (hardware) safety and control system (software) safety. Regarding robot hardware safety, limit blocks can be used for mechanical protection. At the same time, the mechanical structure design can fully avoid the invasive design of the robot to the human body by establishing the robot's kinematic model and analyzing the robot's reachable space. Regarding robot software safety, the stability of the existing closed-loop system for the interactive control of rehabilitation robots should be addressed with emphasis due to the dynamical uncertainty of the robot system and the fact that some action conflicts often occur during the physical human-robot interaction. At the same time, the damping response performance of the system should be improved.

### 4.2. Individual Difference Adaptations

Although some studies have been conducted on adaptive aids and postural and motor adaptations, there is a lack of systematic and targeted research. The number of rehabilitation patients is growing annually, and the parameters of the rehabilitation programs vary greatly depending on the individual's posture, degree of motor impairment, pathology, and stage of rehabilitation. In response to individual differences in body shape and pathology, rehabilitation robots should be individualized in configuration, control systems, movement trajectories, training modes, and functional parameters. In this regard, further research should be conducted on the adaptive mechanism of individual and pathological differences, the adaptability and stability of the control system, the application of sensor technology, and the design of control algorithms so that the rehabilitation robot can sense the state information of the patient's force and position and adopt the corresponding training mode, control strategy, and matching training parameters. In addition, the flexibility of the structure should be considered, and modular designs can be utilised to make the structure and size of the rehabilitation robot adaptable to individual differences.

### 4.3. Human-Robot Interactivity

Patients can interact with robots in a secure, pleasant, and natural environment if interaction control is effective. Impedance control and hybrid force/position control are the most common control mechanisms employed in the former case. In the latter, the most widely used strategies are myoelectric signal control and EEG signal control. The principles, advantages, and disadvantages of various interactive control methods are shown in (Table 7). Among them, impedance control and myoelectric signal control significantly affect the flexibility of rehabilitation movements, accurately recognizing patients' movement intentions, improving movement control accuracy, and protecting patients from secondary injuries. In contrast, the brain-computer interface (BCI) control method is more challenging to acquire and extract features because the EEG signal is susceptible to multiple interferences, and EEG is not suitable for patients with brain injury [89–92]. Therefore, the current research on the application of brain-computer interface control is significantly constrained [93, 94]. However, relevant studies on brain-computer interface control have been conducted by the University of Washington [95], a research group in the Netherlands [96], Yongwook et al. [97], and Du et al. [98, 99], all of which have demonstrated the feasibility of brainwave control. It is believed that with the continuous improvement and maturity of the technology, EEG signal control will play a significant role in the control of rehabilitation robots in the future.

In addition to the HCI performance factors mentioned above, HCI strategies can also affect the HCI experience and rehabilitation outcomes. In the case of rehabilitated patients, the weak motor skills of the affected limbs and the lack of motivation to participate in a rigid, boring, and repetitive training paradigm will directly lead to poor rehabilitation efficiency and effectiveness. A robust incentive mechanism is a way forward for human-robot interaction in rehabilitation. Future research should combine rehabilitation robots with virtual reality (VR) technology to integrate task-oriented training paradigms. While exciting and diverse virtual scenarios can effectively increase patients' motivation for rehabilitation training, the immersive VR environment can also effectively stimulate mirror neurons in the motor cortex of the human brain and promote neural recovery.

### 4.4. Evaluation Indicators and Systems

As a typical human-robot interaction system, the human factors evaluation of the rehabilitation robot only stays in the study of human comfort or the performance of the equipment from a single aspect in the human-robot system. It has not yet involved a comprehensive, systematic, and in-depth study of human-robot adaptability issues, such as safety and comfort, human-robot fit, pathological adaptability of movement patterns, and trajectories from the mapping matching relationship of human-robot characteristics and lacks perfect evaluation indexes and evaluation systems. Therefore, future research should effectively integrate psychological, subjective evaluation, and physiological human response parameters to achieve a comprehensive human-robot suitability evaluation of rehabilitation robots using geometrical and functional characteristics as direct inputs, which is essential for optimizing the overall performance of rehabilitation robots, developing more targeted training methods and maximizing human-robot matching.

### 4.5. Limitation

1. On the one hand, human factor engineering research for rehabilitation robots is cross-disciplinary research. In human factor engineering research for rehabilitation robots, there is a one-to-many cross-mapping relationship between human factors goals and rehabilitation robot system design. For example, the realization of the same human factors goal is mapped to multiple levels of rehabilitation robot system design, and the design of the same system level is also mapped to various human factors goals. Therefore, the categories of “high safety implementation,” “lightweight and high comfort implementation,” and “high human-robot interaction implementation” in this study are based on the human factors goals and system levels. The contents of the three classifications will inevitably overlap. On the other hand, these classifications are derived from a series of processes, such as literature search, review, evaluation, and statistics, to arrive at high-frequency terms (as shown in Table 7). Therefore, this classification has a certain theoretical basis and reliability and does not influence the feasibility of this article.
2. The main direction of this article is to review the impact of human factors on rehabilitation robots and their research progress and to make suggestions for further research directions. One of our suggestions for subsequent research directions is “robust safety protection mechanisms.” Then, mechanism design (hardware) safety and control system (software) safety are small dimensional suggestions based on the general direction of “sound safety protection mechanism.”

## 5. Conclusion

This review provides an exhaustive review of research advances in human factor engineering in rehabilitation robotics through a series of methodological processes, including literature search and screening, quality assessment, and data extraction. This study classifies existing human factors research in the context of the rehabilitation robotics field, using specific human factors goals as the classification criteria. Based on the four categories of human factors goals, the research progress in rehabilitation robotics is systematically sorted, and the benefits of the corresponding technical solutions for the practical use of rehabilitation robots are analyzed in detail. This systematic review focuses on the lack or inadequacy of current research in terms of the connotation categories of human factors goals, and suggestions are made for subsequent human factors research.

The application of human factor engineering in rehabilitation robots can guarantee that rehabilitation robots can better replace rehabilitation therapists in providing patients with safe, comfortable, and efficient human-robot interaction training. Under the scope of human factor engineering connotation, this study innovatively provides information on human factors goals and technical solutions related to rehabilitation robots, emphasizing the benefits, challenges, goals, and recommendations for the application of human factors in rehabilitation robots, further improving the scope of human factors research on rehabilitation robots and helping to promote rehabilitation robots to maximize the “robots fits human.”

Future research should give full attention to the characteristics of human factor engineering in the development of rehabilitation robots and explore the solutions of sound safety protection mechanisms, human-robot interaction incentive mechanisms, individual difference adaptation mechanisms, and human-robot suitability evaluation systems from multiple perspectives, for example, from the dual dimensions of institutional design (hardware) safety and control system (software) safety. For example, the safety protection mechanism can be improved from both institutional design (hardware) safety and control system (software) safety. At the same time, through multidisciplinary collaboration and the combination of various research techniques, the hypotheses and solutions can be verified to enhance the safety, usability, ease of use, and inclusiveness of the rehabilitation robot as a new rehabilitation tool and instrument. This is important for developing a harmonious human-robot relationship, enhancing rehabilitated patients' motor function and quality of life, and assisting rehabilitation robots in gradually becoming practical, mature, and finally achieving large-scale application.

## Figures and Tables

**Table 1:** Three-column search term combination table.

I	II	III	
Rehabilitation Robot Rehabilitation Exoskeleton Exoskeleton Rehabilitation Robots	Human factors engineering Human factors Human characteristics Human ergonomics Ergonomics	Body adaptability Safety Styling comfort Structural comfort Operating comfort Light weight Human-robot relationship Human-robot fit Human-robot interaction Ease of operation Adaptability Motion mode Motion trajectory Motion planning Motion matching Motion coordination Motion flexibility	Flexibility of movement Structural adaptability Styling adaptation Kinematic analysis Kinetic analysis Pathological adaptation Impedance control Coordination control Fatigue Performance evaluation Pressure distribution sEMG EEG Motion capture Virtual simulation Heart rate Human-robot interface

**Table 2:** Quality verification of qualitative studies reviewed.

Studies reviewed	Critical appraisal criteria							Conclusion
	(1)	(2)	(3)	(4)	(5)	(6)	(7)	
Kirby Ann Witte et al	Yes	No	Yes	Yes	Yes	Unclear	No	Reviewer 1: inclusion
	Yes	Yes	Unclear	No	Yes	No	Yes	Reviewer 2: inclusion

Toshiaki Tsuji et al	No	Yes	Yes	Yes	No	Yes	No	Reviewer 1: inclusion
	Yes	No	No	Yes	Unclear	Yes	Yes	Reviewer 2: inclusion

Note. The evaluation criteria for qualitative research are as follows. (1) There is consistency between the stated viewpoint and the research method. (2) There is consistency between the research methods and the research questions or objectives. (3) There is consistency between the research methods and the methods used to collect the data. (4) There is consistency between the research methods and the presentation and analysis of the data. (5) There is consistency between the research methods and the interpretation of the results. (6) The research is ethical according to current standards or, for recent studies, there is evidence of theoretical approval by the appropriate agency. (7) The conclusions in the study are largely derived from the analysis or interpretation of the data.

**Table 3:** Quality verification of the quantitative studies reviewed.

Studies reviewed	Critical appraisal criteria					Conclusion
	Selection bias	Study design	Confounders	Blinding	Data collection method	
Yang Liu et al.	Moderate	Moderate	Weak	Moderate	Strong	Reviewer 1: inclusion
	Weak	Moderate	Moderate	Moderate	Strong	Reviewer 2: inclusion

Moyao Gao et al.	Weak	Moderate	Weak	Moderate	Moderate	Reviewer 1: inclusion
	Moderate	Moderate	Weak	Moderate	Moderate	Reviewer 2: inclusion

Jing Chen et al.	Weak	Moderate	Strong	Moderate	Moderate	Reviewer 1: inclusion
	Weak	Moderate	Moderate	Moderate	Strong	Reviewer 2: inclusion

Siqi Li et al.	Moderate	Moderate	Weak	Moderate	Strong	Reviewer 1: inclusion
	Moderate	Moderate	Weak	Moderate	Strong	Reviewer 2: inclusion

Yanlin Wang et al.	Moderate	Strong	Weak	Moderate	Moderate	Reviewer 1: inclusion
	Moderate	Strong	Weak	Weak	Moderate	Reviewer 2: inclusion

Shahid Hussain et al.	Weak	Moderate	Weak	Moderate	Strong	Reviewer 1: inclusion
	Moderate	Moderate	Weak	Moderate	Strong	Reviewer 2: inclusion

Lili LI et al.	Weak	Strong	Moderate	Moderate	Moderate	Reviewer 1: inclusion
	Weak	Moderate	Moderate	Moderate	Strong	Reviewer 2: inclusion

Qiaolian Xie et al.	Weak	Moderate	Moderate	Strong	Moderate	Reviewer 1: inclusion
	Weak	Moderate	Strong	Moderate	Moderate	Reviewer 2: inclusion

Kai Gui et al.	Moderate	Strong	Weak	Moderate	Moderate	Reviewer 1: inclusion
	Moderate	Strong	Moderate	Moderate	Moderate	Reviewer 2: inclusion

Lorenzo Grazi et al.	Moderate	Weak	Weak	Moderate	Strong	Reviewer 1: inclusion
	Moderate	Moderate	Weak	Moderate	Strong	Reviewer 2: inclusion

Jianfeng Li et al.	Weak	Strong	Moderate	Moderate	Moderate	Reviewer 1: inclusion
	Moderate	Strong	Moderate	Moderate	Moderate	Reviewer 2: inclusion

**Table 4:** Quality verification based on the mixed methods appraisal tool (MMAT) of mixed-method studies reviewed.

Studies reviewed	Critical appraisal criteria													Conclusion
	(1)	(2)	(3)	(4)	(5)	(6)	(7)	(8)	(9)	(10)	(11)	(12)	(13)	
Lizheng Pan et al.	Yes	No	No	Yes	Yes	Yes	No	Yes	Yes	Yes	Yes	No	Yes	Reviewer 1: inclusion
	Yes	Yes	Yes	No	Yes	Yes	No	Yes	Yes	Yes	Yes	No	Yes	Reviewer 2: inclusion

Jian Li et al.	Yes	No	No	Yes	Yes	Yes	Yes	No	Yes	Yes	Yes	No	Yes	Reviewer 1: inclusion
	Yes	Yes	No	Yes	Yes	Yes	No	No	Yes	Yes	Yes	No	Yes	Reviewer 2: inclusion

Junlin Wang et al.	Yes	No	Yes	Yes	Yes	No	Yes	No	Yes	Yes	Yes	Yes	No	Reviewer 1: inclusion
	Yes	No	Yes	Yes	Yes	No	Yes	No	Yes	No	Yes	Yes	Yes	Reviewer 2: inclusion

Can Wang et al.	Yes	Yes	No	Yes	Yes	No	Yes	No	Yes	Yes	Yes	No	Yes	Reviewer 1: inclusion
	Yes	Yes	No	Yes	Yes	No	Yes	No	Yes	Yes	Yes	Yes	No	Reviewer 2: inclusion

Yanlin Wang et al.	Yes	Yes	Yes	No	No	Yes	No	Yes	Yes	Yes	No	Yes	Yes	Reviewer 1: inclusion
	Yes	Yes	Yes	Yes	No	Yes	No	Yes	No	Yes	No	Yes	Yes	Reviewer 2: inclusion

Stefano Marco Maria De Rossi et al.	Yes	Yes	No	Yes	No	Yes	Yes	Yes	No	Yes	No	Yes	Yes	Reviewer 1: inclusion
	Yes	Yes	No	Yes	No	Yes	Yes	Yes	No	Yes	No	Yes	No	Reviewer 2: inclusion

Leiyu Zhang et al.	Yes	Yes	Yes	No	Yes	Yes	No	Yes	No	Yes	Yes	Yes	No	Reviewer 1: inclusion
	Yes	Yes	Yes	No	Yes	Yes	No	Yes	No	Yes	Yes	Yes	No	Reviewer 2: inclusion

Christian Di Natali et al.	Yes	Yes	Yes	No	Yes	No	Yes	Yes	Yes	Yes	No	Yes	Yes	Reviewer 1: inclusion
	Yes	Yes	Yes	No	Yes	No	Yes	No	Yes	Yes	No	Yes	No	Reviewer 2: inclusion

Note. The criteria for combining qualitative and quantitative critical evaluation are as follows. (1) Is the qualitative approach appropriate for the research question? (2) Are the qualitative data collection methods adequate to address the research question? (3) Are the results of the study adequately derived from the data? (4) Are the interpretations of the results adequately supported by the data? (5) Is qualitative data sources, collection, analysis, and interpretation coherence? (6) Is the sampling strategy relevant to address the research question? (7) Is the sample representative of the target population? (8) Are the measurements appropriate? (9) Is the statistical analysis appropriate for solving the research question? (10) Is there a good rationale for using a mixed-methods design to address the research question? (11) Are the different parts of the study effectively integrated to answer the research questions? (12) Are the integration results of the qualitative and quantitative components adequately explained? (13) Are disagreements and inconsistencies between quantitative and qualitative results adequately addressed?

**Table 5:** Characteristics of the studies reviewed.

General characteristics		Document		Study design			Method(s)				Number of participants
Author(s)	Year of publication	Journal article	Conference paper	Quantitative	Qualitative	Mixed-methods	Experimental	Questionnaire surveys	Simulation experiment	Real experiment	
Pan et al. [22]	2013	√				√	√		√	√	6
Li et al. [23]	2019	√				√	√		√		4
Liu et al. [24]	2021	√		√			√			√	5
Ann Witte et al. [25]	2017		√		√		√		√		3
Wang et al. [26]	2018	√				√	√			√	9
Gao et al. [27]	2022	√		√			√		√	√	7
Chen et al. [28]	2020	√		√			√			√	5
Wang et al. [29]	2021	√				√	√			√	6
Li et al. [30]	2019	√		√			√			√	4
Wang et al. [31]	2020	√		√			√			√	4
Hussain et al. [32]	2016	√		√			√			√	4
Li et al. [33]	2017	√		√			√		√	√	5
Xie et al. [34]	2021	√		√			√			√	6
Gui et al. [35]	2017		√	√			√			√	3
Wang et al. [36]	2020	√				√	√		√		5
De Rossi et al. [37]	2011	√				√	√			√	10
Grazi et al. [38]	2016	√		√			√			√	6
Zhang et al. [39]	2019	√				√	√			√	6
Di Natali et al. [40]	2020	√				√	√		√	√	4
Tsuji et al. [41]	2016	√				√	√			√	3
Li et al. [42]	2021	√			√		√			√	3

**Table 6:** Human factors targets characteristics of the included studies.

Author (s)	Human factors targets											
	Safety	Lightness	Comfort	Suppleness and stability	Coordination	Adaptive and on-demand assistance	Safety evaluation	Comfort evaluation	Sport performance evaluation			Ergonomics analysis and evaluation
									Stability	Matchability	Compatibility	
Pan et al. [22]	√			√					√			
Li et al. [23]	√			√					√			
Liu et al. [24]		√										
Ann Witte et al. [25]		√	√									
Wang et al. [26]		√	√									
Gao et al. [27]			√	√					√	√		
Chen et al. [28]	√			√					√			
Wang et al. [29]				√					√			
Li et al. [30]				√					√			
Wang et al. [31]	√			√					√			
Hussain et al. [32]						√						
Li et al. [33]						√						
Xie et al. [34]					√				√	√		
Gui et al. [35]					√							
Wang et al. [36]							√					
De Rossi et al. [37]								√				
Grazi et al. [38]								√				
Zhang et al. [39]									√			
Di Natali et al. [40]									√			
Tsuji et al. [41]										√		
Li et al. [42]			√								√	√

**Table 7:** Principles and advantages and disadvantages of various types of interactive control methods.

Types of control	Interactive control based on force information		Interactive control based on bioelectrical signals	
Control strategy	Impedance control	Hybrid force/position control	Electromyography interaction	Brain-computer interaction
Principle	The control object is the dynamic relationship between force and position	Signal command with a deviation of position and deviation of force/torque	Electrical signals acquired through EMG sensor measurements	Acquisition of EEG signals that express the patient's motor thinking

Advantages	Active suppleness and stable synergy can be achieved	The accuracy is high and the control performance is more reliable and stable	High sensitivity can accurately reflect the intention of human movement and interactive control flexibility	Real-time, more rapid recognition of motion intent

Disadvantage	A large number of sensors need to be installed to detect force and position information in real-time	Accurate modelling is needed, and the real-time requirements for the system are high	The sEMG signal is weak and has a large degree of instability when measured	The EEG signal is poorly resistant to interference and has poor stability performance

Application suggestions	It is mainly used in active training mode to better reflect the patient's subjective motor intention and achieve more reliable, stable and supple motor control		It is mainly used for patients with weak voluntary mobility of the affected limb to achieve master-slave control of the affected limb by the healthy limb	Not limited to the degree of physical disability, but not for patients with brain injury
